# RNA binding protein serine/arginine splicing factor 1 promotes the proliferation, migration and invasion of hepatocellular carcinoma by interacting with RecQ protein-like 4 mRNA

**DOI:** 10.1080/21655979.2021.1972785

**Published:** 2021-09-04

**Authors:** Ying Ye, Feng Yu, Zhao Li, Yaping Xie, Xiaohong Yu

**Affiliations:** Wuchang Hospital Affiliated to Wuhan University of Science and Technology, Wuhan, Hubei, China

**Keywords:** RECQL4, RNA binding protein, srsf1, hepatocellular carcinoma, migration

## Abstract

Abnormally high expression of RecQ protein-like 4 (RECQL4) has been observed in many cancers, including hepatocellular carcinoma (HCC). We aimed to explore the effects of RECQL4 on HCC progression and the possible mechanisms. RECQL4 expression in HCC tissues and its correlation with the prognosis of HCC patients were analyzed using GEPIA2 and UALCAN databases. After detecting RECQL4 levels in several human HC cell lines, RECQL4 was silenced by siRNA transfection. Cell viability, migration and invasion were tested with CCK-8, wound healing and transwell assays. The levels of epithelial–mesenchymal transition (EMT) proteins were evaluated by western blotting. The ENCORI database was adopted for the analysis of the correlation between RECQL4 and serine/arginine splicing factor 1 (SRSF1) in HCC tissues. RNA immunoprecipitation and actinomycin D addition assay were employed to evaluate the combination of these two genes. SRSF1 was overexpressed to assess the biological function of HCC cells with RECQL4 silencing. Results suggested that RECQL4 was overexpressed in HCC tissues and cell lines, which was related to poor prognosis of HCC patients. RECQL4 loss-of-function repressed the proliferation, migration, invasion and EMT of HCC cells. RECQL4 was positively correlated with SRSF1 in HCC tissues. Moreover, SRSF1 was confirmed as an RNA binding protein of RECQL4. Further experiments found that SRSF1 knockdown reduced the stability of RECQL4 mRNA. Rescue assays indicated that SRSF1 overexpression crippled the braking effects of RECQL4 knockdown on the progression of HCC cells. Collectively, SRSF1 can bind to RECQL4 mRNA and enhance its stability, thereby promoting the progression of HCC.

## Introduction

Hepatocellular carcinoma (HCC), one of the most common malignant neoplasms in clinical practices, is the leading cause of cancer-associated mortality [1,2]. Despite advancements in the early detection and surgical operations of HCC, there are still many HCC patients diagnosed at advanced stages by the time of initial diagnosis [3,4]. Therefore, it is urgent to deeply elucidate the molecular mechanism of HCC and identify promising targets for therapeutic intervention for HCC.

RecQ protein-like 4 (RECQL4), one of the five human RecQ family of DNA helicases, is a guardian of genome stability accountable for repairing DNA by controlling the initiation of DNA replication, telomere maintenance and repair of DNA double-strand breaks [5,6]. A considerable body of evidence indicates that RECQL4 is elevated in a large number of malignant tumors, such as ovarian cancer, gastric cancer and breast cancer [7–9]. In HCC, RECQL4 is found to be highly expressed and can be used as a diagnostic marker for the prognosis of this disease [10]. However, the effects of RECQL4 on the progression of HCC and the possible regulatory mechanisms remain to be reported.

RNA binding proteins (RBPs) are a class of proteins that bind to coding or noncoding transcripts, which can regulate RNA expression through mediation of RNA splicing, mRNA stability and localization and translation efficiency, thereby playing a crucial role in posttranscriptional control of gene expression [11]. An increasing number of researchers have validated that RBPs are implicated in the expression of different genes responsible for biological activities, whereas unexpected mutations of RBPs can trigger cancer progression [12,13]. Serine/arginine splicing factor 1 (SRSF1) is a key splicing factor that functions in constitutive and alternative splicing [14]. In addition to splicing regulation, SRSF1 was demonstrated to be involved in various biological functions, such as RNA transport, translation control and senescence [15,16]. As an important RBP, SRSF1 regulates the alternative splicing and stability of mRNAs such as LIG1 and PTPMT1, and promotes the malignant progression of tumors [17,18].

In this study, we started from the detection of RECQL4 expression in HCC tissues and cell lines to the analysis of RECQL4 on the proliferation, migration and invasion of HCC cells. Moreover, the potential mechanism of RECQL4 expression regulated by SRSF1 I HCC was investigated. Our findings may be conducive to the exploration into therapeutic targets for HCC.

## Materials and methods

### Cell culture

Several human HCC cell lines (Hep10, HuH-7, SNU-387 and Li-7) and human immortalized liver cell line MIHA were provided by the cell bank of Chinese Academy of Sciences (Shanghai, China). Cells were grown in Dulbecco’s modified Eagle’s medium (DMEM; Gibco, Grand Island, USA) supplemented with 10% FBS (Gibco). Cells were kept in a humidified atmosphere containing 5% CO_2_ at 37°C.

### Bioinformatics analysis

The expression of RECQL4 in HCC tissues and the correlation between RECQL4 and the prognosis of patients with HCC were analyzed using Gene Expression Profiling Interactive Analysis (GEPIA) 2 (http://gepia2.cancer-pku.cn/#degenes) and UALCAN (http://ualcan.path.uab.edu/index.html) databases. The correlation between RECQL4 and SRSF1 expression in HCC tissues as well as the interaction between RECQL4 and SRSF1 were analyzed with ENCORI database. The binding sequences between RECQL4 and SRSF1 were predicted by PRIdictor (http://pridb.gdcb.iastate.edu/RPISeq/results.php).

### Cell transfection

The small interfering RNAs (siRNAs) against RECQL4 (si-RECQL4#1 and si-RECQL4#2) or SRSF1 (si-SRSF1#1 and si-SRSF1#2), the negative control siRNA (si-NC), SRSF1 pcDNA3.1 plasmid (Oe-SRSF1) and the empty vector plasmid (Oe-NC) were obtained from RiboBio (Guangzhou, Guangdong, China). All transfections were conducted with Lipofectamine 2000 (Invitrogen, Carlsbad, CA, USA) according to the product instructions. After 48 h, cells were collected, and the expression of RECQL4 or SRSF1 was monitored with RT-qPCR and western blotting.

### Cell viability assay

The viability of HuH-7 cells was examined with a Cell Counting Kit-8 kit (CCK-8; Dojindo, Japan). Cells (at a density of 3 × 10^3^ cells/well) were plated into 24-well plates and cultured for adherence. For each well, CCK-8 reagent of 10 μl was added. After the cells were maintained at 37°C for 2 h, the absorbance was measured at 450 nm.

### Wound healing assay

2 × 10^5^ HuH-7 cells were plated into 6-well plates and cultured until 95% confluence. Then, serum-free DMEM was employed to incubate cells overnight. The cell layer was wounded by scraping with a sterile plastic tip. After incubation for 24 h at 37°C in completed DMEM, the debris was eluded by phosphate buffer saline. Quantification of cell motility was conducted by subtracting the wound width at each time point from the wound width at the 0 h time point.

### Transwell cell invasion assay

The ability of HuH-7 cell invasion was evaluated by 24-well transwell chambers with 8 μm pores provided by Corning (NY, USA) inserts pro-coated with Matrigel (BD Biosciences, Franklin Lakes, NJ, USA). 5 × 10^4^ HuH-7 cells suspended in 200 µl of serum-free DMEM were seeded into the upper chamber. Then, 600 µl DMEM containing 10% FBS was filled in the lower chamber. After 24 h, cells attached to the lower surface were fixed with 4% paraformaldehyde and stained with crystal violet. The invaded cells were observed under an inverted light microscope, and the number was counted in five randomly different fields.

### RNA immunoprecipitation (RIP) assay

An EZ Magna RIP kit (Millipore, Billerica, MA) was utilized to analyze the combination between RECQL4 and SRSF1 in HuH-7 cells. Briefly, cells (1 × 10^7^) were lysed in the RIP Lysis Buffer. Subsequently, the cell lysates underwent incubation at 4°C, with magnetic beads conjugating the antibodies against SRSF1 or negative control IgG for 3 h. The immunoprecipitated RNA was collected, and the RECQL4 expression was analyzed with RT-qPCR.

### Actinomycin dassay

After transfection, HuH-7 cells were exposed to 5 μg/mL actinomycin D (ActD; Sigma-Aldrich), followed by incubation for 2, 4, 6, or 8 h. The mRNA stability of RECQL4 in the HuH-7 cell exposed to ActD was determined with RT-qPCR.

### RT-qPCR

Isolation of total RNA from HCC cells was performed by means of TRIzol® regent (Invitrogen; Thermo Fisher Scientific, Inc.) following the manufacturer’s recommendations. Afterward, a PrimeScript RT Reagent kit (Promega Corporation) was utilized for synthesis of complementary DNA. PCR analysis of the gene expression was carried out on an ABI 7500 thermocycler (Applied Biosystems) with SYBR-Green PCR Master Mix (Thermo Fisher Scientific, Inc.). Housekeeping gene GAPDH was used as the internal control for normalization. The relative mRNA expression was calculated with the 2^−ΔΔCq^ method [19].

### Western blot analysis

Total protein extracts were obtained by RIPA lysis buffer (Beyotime) and then quantified by a bicinchoninic acid kit (Beyotime). Of each sample, an equal amount of each sample (40 μg) was resolved on 10% sodium dodecyl sulfate-polyacrylamide gel electrophoresis and transferred onto nitrocellulose membranes. Possible nonspecific binding was blocked by 5% skim milk and then incubated overnight at 4°C with the corresponding primary antibodies. These blots were then incubated with horseradish peroxidase conjugated secondary antibody for 2 h. These immunoreactive bands underwent enhanced chemiluminescence exposure (Life Technologies, Pleasanton, CA, USA). Band densities of target proteins were quantified using Image J software and normalized to GAPDH.

### Statistical analysis

All experiments were performed with three biological replicates. The statistical significance between the two comparator groups was ascertained with Student’s *t*-test. The comparison employed one-way analysis of variance followed by Tukey’s post hoc test for multiple groups. For all the tests p values <0.05 was considered statistically significant.

## Results

### RECQL4 is highly expressed in HCC tissues and cell lines and is related to the poor prognosis

**Figure 1.**
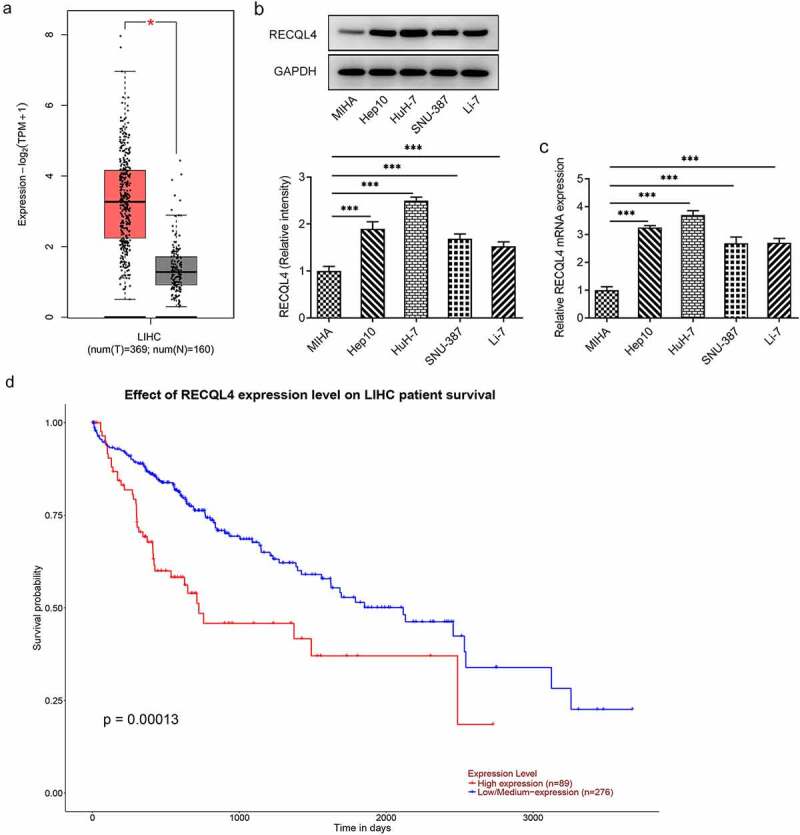
Highly expressed RECQL4 in HCC tissues and cell lines was related to the poor prognosis. (a) GEPIA2 database was employed to analyze RECQL4 expression in HCC tissues (t) and normal liver tissue (n). (b-c) RECQL4 expression in several HCC (Hep10, HuH-7, SNU-387 and Li-7) cell lines and one human immortalized liver cell line MIHA was examined with western blot analysis and RT-qPCR. (d) the relationship between RECQL4 and survival probability was evaluated using UALCAN database. *P < 0.05, ***P < 0.001

A growing body of literature has shown that RECQL4 is highly expressed in various malignant tumors, such as ovarian cancer, gastric cancer and breast cancer [7–9]. To explore the role of RECQL4 in HCC, GEPIA2 database was employed to analyze RECQL4 expression in HCC tissues (T) and normal tissues (N). In comparison to the normal tissues, a remarkably upregulated RECQL4 expression was observed in the tumor tissues (Figure 1(a)). Further results of western blotting and RT-qPCR exhibited in (Figure 1(b-c)) revealed that RECQL4 expression was elevated in the HCC cell lines (Hep10, HuH-7, SNU-387 and Li-7) when compared to the human immortalized liver cell line MIHA, and the highest RECQL4 level was found in HuH-7 cells. Therefore, HuH-7 cell line was chosen to implement the subsequent experiments. Additionally, the UALCAN database indicated that higher expression of RECQL4 displayed the lower survival probability in patients with HCC (Figure 1(d)). These results imply the potentially important role of RECQL4 in HCC.

### RECQL4 deletion inhibits the proliferation, migration and invasion of HCC cells

**Figure 2.**
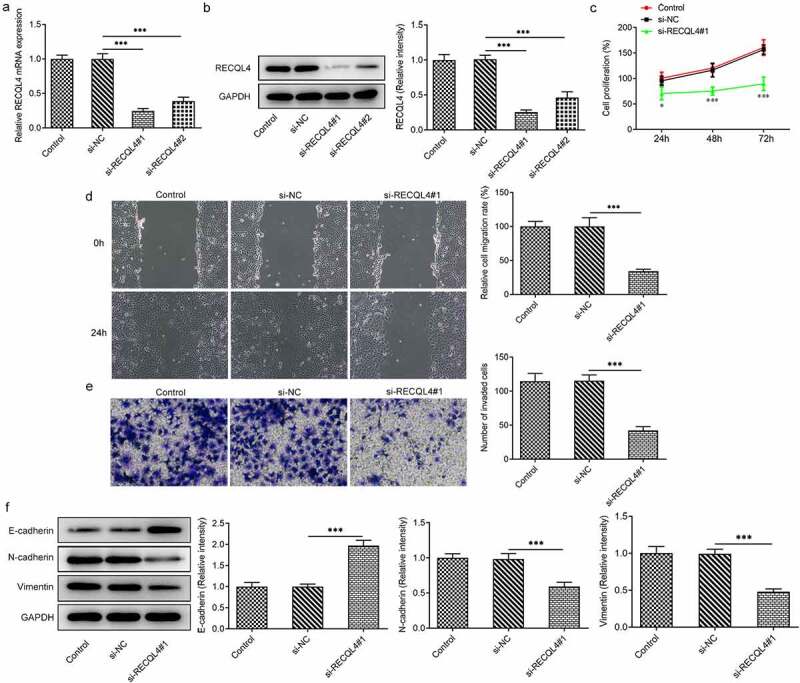
RECQL4 deletion inhibited the proliferation, migration and invasion of HCC cells. (a-b) RT-qPCR and western blot analysis were utilized for detection of RECQL4 expression after transfection with si-RECQL4#1 or si-RECQL4#2 in HuH-7 cells. ***P < 0.001. (c) cell viability was assessed with CCK-8 assay. *P < 0.05, ***P < 0.001 vs. si-NC. (d) the capacity of cell migration was tested by wound healing assay. (e) transwell assay was adopted for determining cell invasion. (f) the expression of E-cadherin, N-cadherin and vimentin was tested with western blotting. ***P < 0.001

Afterward, RECQL4 was silenced by transfection with si-RECQL4#1 or si-RECQL4#2 in HuH-7 cells to investigate the effects of RECQL4 on the progression of HCC. As what is observable from Figure 2(a-b), si-RECQL4#1 or si-RECQL4#2 led to notably decreased RECQL4 expression levels compared with the si-NC group. A better interference efficiency was noticed subsequent to transfection by si-RECQL4#1, which was used to conduct further experiments. Results in CCK-8 assay suggested that RECQL4 silencing conspicuously inhibited cell viability relative to the si-NC group (Figure 2(c)). In addition, as shown in (Figure 2(d)), deletion of RECQL4 clearly decreased the migratory ability of HuH-7 cells when compared to the si-NC group. Meanwhile, the effect of RECQL4 knockdown on cell invasion was consistent with the outcome from the wound healing assay (Figure 2(e)). Our study also investigated the contribution of RECQL4 to expression of epithelial–mesenchymal transition (EMT) related proteins, which are crucial for the migration and invasion of malignant cells. It was found that si-RECQL4#1 transfection obviously upregulated the E-cadherin expression but downregulated the N-cadherin and vimentin expressions in HuH-7 cells related to the untreated transfected group (Figure 2(f)). These data provide evidence that RECQL4 deletion represses the proliferation, migration and invasion of HCC cells.

### SRSF1 is a RNA binding protein of RECQL4 and can regulate RECQL4 expression

**Figure 3.**
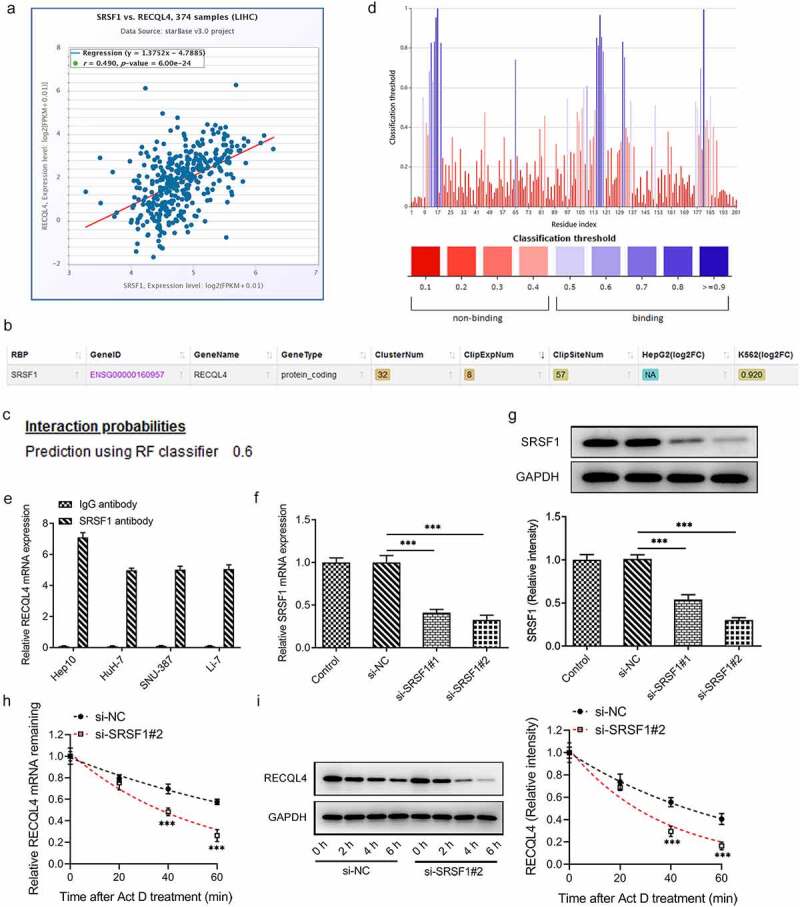
SRSF1 was a RNA binding protein of RECQL4 and could regulate RECQL4 expression. (a) the correlation between RECQL4 and SRSF1 was analyzed by ENCORI database. (b) the binding of SRSF1 to RECQL4 mRNA was predicted with ENCORI database. (c) the combination probability of SRSF1 to RECQL4 mRNA was analyzed using database. (d) the binding sites of SRSF1 to RECQL4 mRNA were analyzed by means of predictor. (e) RIP assay was performed to verify the combination between SRSF1 and RECQL4. (f-g) SRSF1 mRNA and protein levels after transfection with si-SRSF1 were determined with RT-qPCR and western blotting. (h-i) SRSF1 mRNA and protein levels after HuH-7 cells exposed to actinomycin D were determined with RT-qPCR and western blotting. **P < 0.01, ***P < 0.001 vs. si-NC

To elucidate the potential mechanisms of RECQL4 in the progression of HCC, the correlation between RECQL4 and SRSF1, an important RBP, was analyzed using the ENCORI database. As presented in (Figure 3(a)), there was a positive correlation between RECQL4 and SRSF1 expression in HCC tissue samples. Additionally, the ENCORI database also predicted that SRSF1 could serve as an RBP binding to RECQL4 mRNA (Figure 3(b)). Moreover, the combination probability of SRSF1 to RECQL4 mRNA was checked in the PRIDB database, and it was found that the combination probability between them was 0.6 (>0.5 indicated that they could be combined) (Figure 3(c)). Furthermore, PRIdictor predicted the binding sites of SRSF1 to RECQL4 mRNA (Figure 3(d)). The RIP experiment results displayed in (Figure 3(e)) confirmed that SRSF1 could interact with RECQL4 mRNA. Subsequently, SRSF1 was silenced to evaluate the stability of SRSF1 to RECQL4 with actinomycin D assay. RT-qPCR and western blot analysis indicated the reduced SRSF1 expression subsequent to transfection by si-SRSF1#1 or si-SRSF1#2 (figure 3(f-g)). And si-SRSF1#2 was selected for the following actinomycin D assay due to its lower SRSF1 expression. It has been observed that SRSF1 silencing could weaken the stability of RECQL4 mRNA (Figure 3(h-i)). These observations reveal that SRSF1, as an RBP, binds to and enhances the stability of RECQL4 mRNA and promotes the translation of RECQL4.

### SRSF1 overexpression cripples the repressive impact of RECQL4 silencing on the proliferation, migration and invasion of HCC cells

**Figure 4.**
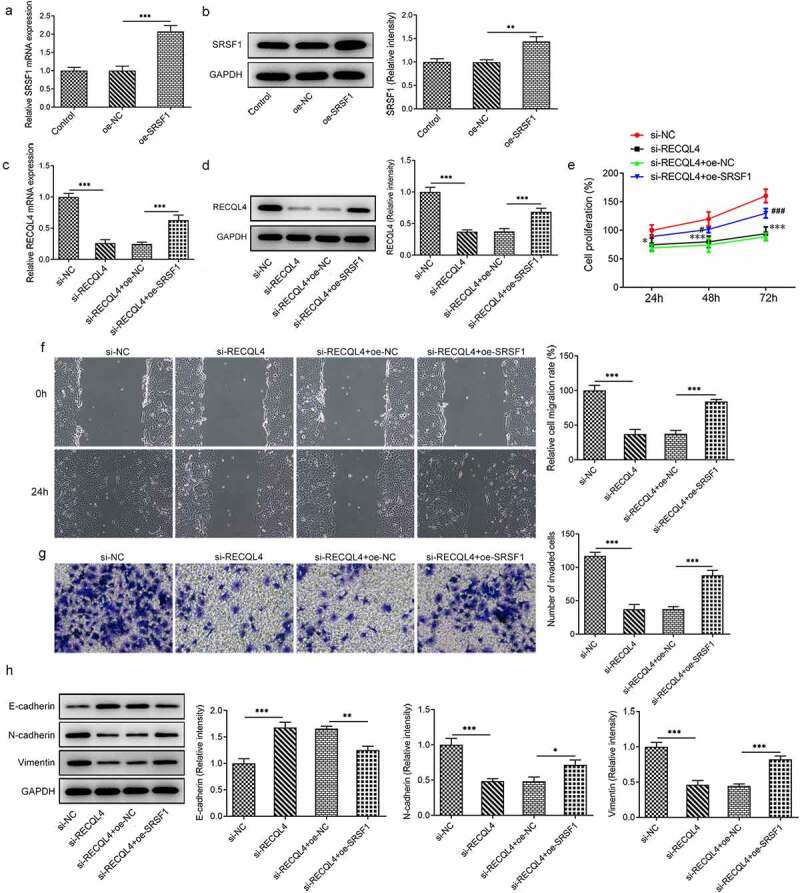
SRSF1 overexpression crippled the repressive impact of RECQL4 silencing on the proliferation, migration and invasion of HCC cells. (a-b) RECQL4 levels were assessed using RT-qPCR and western blot assay after transfection with RECQL4 plasmid. (c-d) RT-qPCR and western blotting were conducted to assess RECQL4 expression after transfection with si-RECQL4 and Oe-SRSF1. **P < 0.01, ***P < 0.001. (e) cell viability was tested with CCK-8 kit. *P < 0.05, ***P < 0.001 vs. si-NC; ^#^P < 0.05, ^###^P < 0.001 vs. si-RECQL4+ oe-NC. (f) the capacity of cell migration was tested by wound healing assay. (g) transwell assay was adopted for measuring cell invasion. (h) the expression of E-cadherin, N-cadherin and vimentin was tested with western blotting. *P < 0.05. **P < 0.01, ***P < 0.001

To further clarify whether SRSF1 contributes to the inhibitory effects of RECQL4 on the properties of HCC cells, SRSF1 was overexpressed by transfection with SRSF1 plasmid. Notably enhanced SRSF1 levels were found in the Oe-SRSF1 group as compared to the empty vector group (Figure 4(a-b)). Then, as what is observable from (Figure 4(c-d)), loss-of-function of RECQL4 dramatically downregulated SRSF1 expression when compared to the si-NC group, which was recovered by SRSF1 overexpression. Additionally, deletion of RECQL4 suppressed the proliferation of HuH-7 cells, whereas SRSF1-upregulation resulted in the proliferation recovery of cells (Figure 4(e)). Besides, the gain-of-function of SRSF1 in RECQL4 silencing HuH-7 cells noticeably restored the migration and invasion repression caused by RECQL4 as gauged by wound healing and transwell assays (Figure 4(f-g)). As expected, SRSF1 overexpression partially counteracted the upregulated E-cadherin and downregulated N-cadherin and vimentin expression triggered by RECQL4 knockdown in HuH-7 cells. Through the above findings, we proved that SRSF1 promotes the progression of HCC by interacting with RECQL4 mRNA.

## Discussion

HCC, a lethal malignant digestive system cancer, is the leading cause of cancer-related deaths worldwide [20]. It is broadly recognized as a cancer with poor prognosis mainly as a result of high tumor recurrence and metastasis [21]. Therefore, to elucidate the metastatic mechanism underlying HCC is imperative. This study showed that RECQL4 expression is remarkably elevated in HCC tissues and cell lines, and what follows the phenomenon of higher RECQL4 level is the worse prognosis of HCC. Additionally, we demonstrated that RECQL4 deletion, whose functions were regulated and stabilized by SRSF1, inhibits the proliferation, migration and invasion of HCC cells.

HCC is a serious malignant tumor characterized by metastasis [22]. Migration and invasion are recognized as two key factors contributing to the aggressiveness and recurrence of HCC [23,24]. EMT is a crucial pathophysiological event in the process of cancer-related migration and invasion [25]. When EMT occurs, epithelial cells lose their tight connections with other cells and gradually acquire mesenchymal cell-like capabilities, leading to the ability of cells to break free of the membrane barrier of tissue [26]. During the course of EMT, epithelial markers, such as E‐cadherin, is elevated, whereas mesenchymal markers such as E-cadherin and vimentin are reduced [27]. RECQL4 is a member of the RECQ helicase family, which plays a significant role in maintaining the stability of nuclear and mitochondrial genomes [28,29]. In recent years, abnormal high RECQL4 expression has been reported in numerous cancers by a body of studies. For instance, in ovarian cancer, RECQL4 can be negatively regulated by miR-10a-5p, thereby promoting cell proliferation and invasion [7]. In a subsequent glioma study, Sylwia K et al. found that upregulated RECQL4 expression, which is closely related to poor survival of glioblastoma patients, accelerates the proliferation of glioma cells [30]. Specifically, another important observation, discovered already in the previous study on prostate cancer, was that RECQL4 may be a new biomarker for advanced prostate cancer due to its increase in metastatic prostate cancer cells and tumor tissues [31]. RECQL4 can also promote the proliferation, migration and EMT of esophageal squamous cell carcinoma [32]. It is worthy of note that previous lines of evidence have indicated the potential of RECQL4 to be a diagnostic marker for the prognosis of HCC [10]. The present study is the first to explore the effects of RECQL4 on the progression of HCC in HCC cells and further consolidated previous posits. In agreement with the aforementioned previous research, our findings demonstrated for the first time that RECQL4 was apparently upregulated in HCC tissues and cell lines, and RECQL4 loss-of-function sustained the proliferation, migration, invasion and EMT of HCC cells.

Accumulating studies have confirmed that RBPs participate in multiple biological activities, such as cell growth, migration, differentiation and invasion, and unexpected mutations of RBPs can trigger cancer progression [33,34]. SRSF1 is a prototypical family member of SR proteins. As an important RBP, SRSF1 not only plays a central role in constitutive and alternative splicing but also regulates other aspects of RNA metabolism, including mRNA stability, nuclear output and RNA transport, and senescence [16,35,36]. SRSF1 can regulate the alternative splicing and stability of mRNAs such as LIG1 and PTPMT1 and promote the malignant progression of tumors [17,18]. Importantly, emerging evidence supports the notion that SRSF1 expression is notably upregulated in HCC [37]. Another study suggested that SRSF1 promotes the migration of HCC cells, indicating the potential regulatory effect of SRSF1 on HCC progression [38]. Our study further revealed that there was a positive correlation between RECQL4 and SRSF1 expression in HCC tissue samples. SRSF1 could serve as an RBP binding to RECQL4 mRNA, maintain the stability of RECQL4 mRNA, and promote the translation of RECQL4. Further SRSF1 loss-of-function experiments demonstrated that SRSF1 overexpression blocked the repressive impact of RECQL4 silencing on the proliferation, migration and invasion of HCC cells, suggesting that SRSF1 could bind to RECQL4 mRNA thereby affecting the progression of HCC.

## Conclusion

To conclude, we find that SRSF1 regulates the proliferation, migration and invasion of HCC cells through modulating RECQL4 by bind to RECQL4 mRNA. This finding provides the first evidence of the interaction between SRSF1 and RECQL in HCC and improves the understanding of the mechanisms involved in the progression of HCC. The current findings provide a possible target for HCC treatment in the future.

## Data Availability

All data generated or analyzed during this study are included in this published article.
